# Case-Based Clinical Ethics Support – A Description and Normative Discussion of Methodological Issues from the Swedish Perspective

**DOI:** 10.1007/s10730-025-09566-5

**Published:** 2025-10-12

**Authors:** Pernilla Pergert, Mia Svantesson, Cecilia Bartholdson, Anders Bremer, Margareta Brännström, Catarina Fischer Grönlund, Niklas Juth, Joar Björk

**Affiliations:** 1 Department of Public Health and Caring Sciences, Centre for Research Ethics and Bioethics (CRB), Uppsala University, Uppsala, Sweden; 2 Department of Women’s & Children’s Health, Karolinska Institutet, Stockholm, Sweden; 3 Faculty of Medicine and Health, University Health Care Research Center, Örebro University, Örebro, Sweden; 4 Astrid Lindgren Children’s Hospital, Region Stockholm, Stockholm, Sweden; 5 Faculty of Health and Life Sciences, Linnaeus University, Växjö, Sweden; 6 Centre of Interprofessional Collaboration within Emergency care (CICE), Linnaeus University, Växjö, Sweden; 7 Department of Nursing, Umeå University, Umeå, Sweden; 8 Department of Research and Development, Region Kronoberg, Växjö, Sweden

**Keywords:** Clinical ethics support, Ethics case reflection, Mixed-method, Moral case deliberation, Normative discussion, Survey

## Abstract

Clinical Ethics Support (CES) includes various forms of systematic support to deal with ethical challenges in healthcare and case-based CES (C-CES) is used for CES in particular cases. The aim was to describe and normatively discuss organizational and methodological aspects of C-CES used in Swedish healthcare. A mixed-methods approach was used. A descriptive survey was answered regarding eight organizations on hospital, regional and national level, with large variations in the number of conducted C-CES activities. Data were compiled and frequencies calculated. Based on the survey results, normative questions were formulated. Six participants, with expertise of C-CES, participated in a normative group discussion. Field notes and transcribed data were analysed qualitatively. The top ranked goal of C-CES was “Supporting decision making”. Mainly prospective cases were used and C-CES was carried out as un-planned and pre-planned sessions. The normative results showed the importance of avoiding making C-CES unattractive to clinicians, for instance by keeping the time frame. The professional backgrounds of C-CES leaders varied greatly and arguments were provided for the *facilitating* role and that C-CES leaders ought not facilitate where they have been clinically engaged. Identified challenges included variations in uptake of C-CES activities that do not mirror the ethical challenges of the context. The unfair uptake of C-CES can be compared with the uptake in Norway where there are legal requirements for CES. In this study patients and families were not reported to request or attend C-CES. Thus, further research and interventions are needed to ensure their representation in Swedish C-CES.

**Supplementary Information:**

The online version contains supplementary material available at 10.1007/s10730-025-09566-5.

## Introduction

Clinical Ethics Support (CES) is an overall term for various forms of organized and systematic support to stakeholders in healthcare organizations as these deal with ethical challenges (Dauwerse et al., 2013; Rasoal et al., 2017). CES has been dubbed “a complex intervention” (Schildmann et al., 2019a) where complexities extend beyond the ethical quandaries themselves to different expectations from personnel of various professions/disciplines, patients, and families (Finder & Bartlett, 2024). Differences in organizational set-up, individual ethics consultants/facilitators’ skills, approaches and methods used, adds further to the complexity (Jakobsen et al., 2024a; Schildmann et al., 2019). Some CES organizations work mainly by providing expertise opinions by clinical ethics committees or individual ethics consultants (Brierley et al., 2021), whereas others facilitate groups of personnel in dealing with ethically challenging patient situations (Schildmann et al., 2019). There are many terms for the latter form of intervention, with terminology differences sometimes denoting important methodological differences and sometimes not. Interventions to support personnel with challenging patient situations include Moral Case Deliberation (Haan et al., 2018; Molewijk et al., 2008; Tan et al., 2018), ethics rounds (Hansson, 2002; Silén et al., 2016; Svantesson et al., 2008), Ethics Case Reflection rounds (Bartholdson et al., 2014, 2018; Meyer-Zehnder et al., 2021), inter-professional ethics communication (Wälivaara et al., 2023) and Norwegian ethics reflection groups (Jakobsen et al., 2024b; Lillemoen & Pedersen, 2015). In the following, we will use the term case-based CES (C-CES) as an umbrella term for CES interventions where a leader helps a group of healthcare personnel (sometimes patients and families are also invited) to reflect on an ethically difficult situation in a clinical case.

In Sweden, previous research indicates that CES delivery may be different across the country (Rasoal et al., 2017; Svantesson et al., 2018). Being both complex and heterogeneous makes evaluation of C-CES a challenge (Schildmann et al., 2019). A recent survey shows that the availability of CES is heterogenous across the country, with 6/16 responding counties reporting no access to CES (Swedish Association of Local Authorities and Regions, 2020). During an ongoing project, to develop an instrument for evaluating CES based on the European Moral Case Deliberation Outcomes Instrument (Euro-MCD) (de Snoo-Trimp et al., 2020; Svantesson et al., 2014, 2019), a national research team realised the need to understand differences and similarities between C-CES methods used across the country. A special interest group was formed and CES researchers were invited to conduct this study. The aims of this paper were to describe and normatively discuss organizational and methodological aspects of C-CES interventions used in Swedish healthcare.

## Methods

A mixed-methods approach was employed, starting with a quantitative descriptive method, followed by qualitative methodology.

### Context

This study was conducted in Sweden, a country where most healthcare is publicly funded and provided by publicly run healthcare providers, or publicly funded and commissioned but run by private healthcare providers (Blomqvist & Winblad, 2024). Unlike some other countries, there is no legal requirement nor financial incentives for healthcare providers to establish or give access to CES in healthcare organisations. There is, furthermore, no specific training for clinical ethicists. Furthermore, the tasks and ambitions of CES in Sweden vary widely, and although there are networks for staff with ethical competence and/or ethical interest, these networks are informal in character and there is no ambition to standardize the access, responsibilities nor competencies for CES in Sweden. Furthermore, ethical challenges related to individual patients in Swedish healthcare are seldom solved by legal proceedings, and thus the potential for CES to avoid and/or contribute to legal processes in individual cases is largely irrelevant (Bergwall, 2021).

### Data Collection

#### Descriptive Survey

A descriptive survey (Supplementary Information 1) was developed, based on the evaluation tool described by Pedersen et al. (2010) on behalf of the European Clinical Ethics Network. The survey was in English to stick as close as possible to the original and to avoid forward and back translation. All possible respondents were known to be highly proficient in English. The survey included open-ended questions asking for the healthcare context of the respondents’ CES organization, the method and underlying theory of the particular form of C-CES provided by the organization, as well as the professional backgrounds and training of C-CES leaders. The survey also included multiple-choice questions where respondents were asked to choose all alternatives that applied to their C-CES and to rank certain alternatives according to importance. The multiple-choice questions were about the overall goals and underlying normative assumptions of the C-CES; who can initiate and who usually participates in the C-CES; the properties of the ethical issues and (if relevant) exclusion criteria for issues to be brought up; if the C-CES was scheduled; how long it lasted and if it was documented; and the role of the C-CES leader.

Key persons, with a leading or chairing role in various Swedish CES organizations, were purposefully selected to gain national representation and a variation regarding C-CES methods used. Twelve key persons were invited via e-mail to choose one type of C-CES activity (at a time) that they/their organization offered and to fill out the information about that C-CES. If they wanted to report on another form of C-CES activity from their organisation, they were encouraged to fill out a second survey. The key persons were asked to fill out the survey based on their perceptions of the C-CES activity in their organization. Key persons who worked in the same organization were encouraged to fill out the survey together and use one survey for each C-CES. Although some surveys were filled out by two respondents together, each survey represented one form of C-CES being practised somewhere in Sweden. Henceforth, the key persons responding to the survey will be called *respondents*. The compiled answers were discussed in the special interest group and a column with comments were added and used to create the questions for the normative discussion.

#### Normative Discussion Groups

Based on the results from the survey, normative questions were formulated by the last author to gain a deeper understanding of the results from the quantitative descriptive survey, e.g., regarding role of the C-CES leaders, views on impartiality and the distribution/uptake of C-CES. The normative questions focused on areas perceived as of central ethical relevance and/or relevance from the point of view of designing and improving the design of C-CES. Six members of the national research team, including CES-researchers and ethicists from diverse professional backgrounds (philosophers, registered nurses, and physicians) and from various regions of Sweden, were invited to participate in the normative discussion, some of whom had also responded to the descriptive survey. The individuals who participated in the normative discussion group will henceforth be called *participants*. Participants received the questions by email prior to the discussion, which was held digitally on 18th March 2024. The discussion was moderated by the last author and took just over two hours. Field notes were taken, and the discussion was recorded and transcribed verbatim.

### Data Analysis

#### Descriptive Survey

Data were compiled in a table (by the first author), and the answers were colour coded and manually counted (frequency).

#### Normative Discussion Group

Field notes and transcribed data were used for the qualitative analysis, which was inspired by Reflexive Thematic Analysis by Braun et al. (2023). Arguments were explored with a particular attention to normative detail and possible normative consensus/disputes. Member checking with participants was used to ensure that all aspects of the discussion had been understood correctly.

### Ethical Considerations

Information to respondents about the aim of the study, the voluntary nature of the study, that answering/not answering would not affect further collaborations, and that participation could be withdrawn, was provided on the first page of the survey. Possible respondents were informed that answering the survey meant to consent to the data being used by the researchers. Participants in the normative discussion group consented orally to participating and to the recording of the session. No ethical vetting was conducted because the study was not of such a nature that the Swedish law on ethics review applies.

## Results

In the following, the results will be presented under two headings: Descriptive results and Normative results. The descriptive results are presented under three subheadings: *Methods and goals of C-CES*, *Organization of C-CES* and *C-CES leadership*.

### Descriptive Results

There were 11 respondents who answered the survey. One did not respond due to illness. Respondents who reported on the same C-CES activities, and thus answered the same survey together, were counted as one respondent, so that the total number of respondents was eight (= the number of C-CES reported on). The key persons were the ones leading or chairing the CES organizations which, taken together, conducted several hundred actual instances of C-CES throughout Sweden. In most cases, the key persons were also, themselves, C-CES leaders/facilitators. Furthermore, most key persons had themselves trained the C-CES leaders and/or conducted evaluations of their C-CES activity/organization, which informed their answers. The settings for the C-CES activities varied, including three from the university hospital level (Karolinska University Hospital in Stockholm, Sahlgrenska University Hospital in Gothenburg, Skane University Hospital in Malmö/Lund), four at the regional level (Kronoberg—including the Ambulance Service, Uppsala—including Uppsala University Hospital, Västerbotten—including Umeå University Hospital, Örebro—including three hospitals), and one at a national level (the Working Group of Ethics of the Nordic Society of Paediatric Haematology and Oncology). Thus, the represented organizations included regional as well as national organizations and with variations in the number of CES leaders and conducted C-CES.

#### Methods and Goals of C-CES

Most (6/8) CES organizations consistently used one specific method when they performed C-CES. That is, one specific method was used within the organizations, but different methods were used between the organizations. Of those who did not use one specific method, one (1/8) used several different methods and one (1/8) used no specific method. The methods used include the Dilemma method (Molewijk et al., 2008), a modified version of the Dilemma method (Silén & Svantesson, 2022), the six-step model (Førde & Pedersen, 2012), the ‘One to five’ method (Fischer-Grönlund et al., 2021), a modified version of the Actor model (Bischofberger et al., 1991), and the Karolinska model (Bartholdson et al., 2014) based on the Actor model. Despite methodological variation, time used per C-CES was notably similar with 7/8 respondents stating that their C-CES activities take about 60 minutes (*n* = 4/8: 30–60 minutes; *n* = 5/8: 61–90 minutes; two respondents selected both). One (1/8) respondent stated their C-CES usually take about 2.5 hours covering several cases over the course of one session.

Regarding the proximal and distal goals currently aimed at by providing C-CES, respondents’ answers and ranking showed much overlap (Table 1). However, one respondent did not rank their answers.

**Table 1:** Overall goals of the C-CES activity and the number of respondents (representing C-CES organisations) who highlighted the response alternatives and ranked them as top three

Overall goals of the C-CES	Number (*n* = 8) who highlighted the alternative	Number (*n* = 7) who ranked alternative as top three*
Decision making support	7	6
Enhance ethical competence/moral learning	7	5
Promote ethical climate	7	3
Providing protected space and time to voice ethical concerns	5	3
Improving the quality of care	4	3
Team building	3	0
Other: Democratic dialogue	1	0

*One Respondent only Ranked Two Alternatives

#### Organization of C-CES

C-CES was carried out both as-per-needed, i.e. unplanned/ad hoc, and as pre-planned sessions, for instance regular sessions once a month planned before having decided on which case to handle. Out of 8 respondents, 4 stated they provided both forms of services, whereas 2/8 did only ad hoc and 2/8 only pre-planned sessions. All but one (7/8) respondents indicated mainly performing C-CES in prospective cases, i.e. in cases where a final decision on how to deal with the dilemma had not yet been made. One (1/8) instead mainly performed C-CES in retrospective cases and with prospective rated as second. Some (3/8) performed retrospective cases as the second most common type of case. When asked to highlight all that apply, most respondents (7/8) reported having exclusion criteria for the issues handled by C-CES (Table 2). Issues related to personnel conflicts were excluded by most. Also, issues where the main challenge was merely distressing situations or merely strong opinions rather than an ethical conflict situation/dilemma were excluded by a majority. In addition, a majority also excluded purely hypothetical cases. In contrast, some respondents reported that “overarching questions” (3/8) and “general themes” (2/8) were among the top three issues handled by C-CES, and thus not an exclusion criteria.

**Table 2:** Exclusion criteria for the issues handled by C-CES and the number of respondents who highlighted respective criteria

Exclusion criteria	Number (*n* = 8) who highlighted the criteria
Issues related to personnel (related to human resources, HR)	7
“Only” a problematic or distressful situation (not a moral question)	5
Strong opinions rather than moral questions (not prepared to listen but rather using C-CES to convince others)	5
Hypothetical case (none of the participants have personal experience of the case)	5
A theme	3
Overarching question	2

Most respondents (7/8) stated that a variety of stakeholders (registered nurses, assistant nurses, physicians, allied health personnel, psychologists/psycho-social personnel, and healthcare managers at various levels) could request a C-CES, although one CES organization (1/8) performed C-CES at the request of healthcare managers only. Physicians were ranked as the most, or second most common stakeholder group to request C-CES by 7/8 respondents. Free text responses indicated that although assistant nurses were formally welcome to request C-CES in most CES organizations, this group may be underrepresented among those actually requesting C-CES. No CES organization reported that they performed C-CES at the request of patients or family members. Regarding relations between the one/ones requesting the C-CES and the C-CES leaders, most respondents (5/8) reported that C-CES leaders within their organization only performed C-CES as an external service, that is, not at the same ward/unit where they worked as healthcare personnel. However, 3/8 reported that C-CES leaders also performed C-CES at their own workplaces, i.e., among their own close colleagues.

#### C-CES Leadership

There was great consensus regarding the role of the leader in C-CES. Most respondents stated that C-CES leaders in their organizations mainly “Listen, talk; try to understand; search ethics focus” and/or “Clarify, ask questions; specify ethics focus”. Indeed, 7/8 respondents provided answers between level 1 and 5 on a scale from 1 to 10 where 1 represents the least degree of normative input from the C-CES leader during the C-CES, and 10 represents the highest degree of normative input from the leader (Table 3). Hence, the answers indicated that a non-authoritarian, facilitating C-CES leader role was employed.

**Table 3:** Levels of normative input/steering provided by the C-CES leader (Pedersen et al., 2010; Reiter-Theil, 2009), and the number of respondents who rated them as top three

Level	Degree of normative input from the C-CES leader	Number of respondents (*n* = 8) who rated as top 3
1	Listen, talk; try to understand; search ethics focus	6
2	Clarify, ask questions; specify ethics focus	6
3	Interpret, evaluate; change perspectives	5
4	Analyse, argue, compare pros and cons	3
5	Refer to, rely on values/norms	2
…		
10	Insist on or resist against decisions or errors	1

There was great variation in the professional backgrounds of C-CES leaders. Most (7/8) respondents stated that their CES organization had C-CES leaders from various professional backgrounds. Healthcare personnel were most common, but professional ethicists and chaplains were also represented. In terms of formal education there was also great variation. Many C-CES leaders had broad ethical competence based on education in ethics/philosophy, but many had no specific methodological training regarding C-CES. However, some had formal training to become a C-CES leader using a specific method, for instance facilitator training of two plus three days with practice in between, or half a day in-house education in the C-CES method.

### Normative Results

Participants (*n* = 6) uniformly agreed that 60–90 minutes is an appropriate time span for C-CES, and that C-CES leaders should be careful not to overstep this time frame. If the process cannot be finished within this time, it is better to divide the C-CES into two parts as longer running times risk making C-CES less attractive in the eyes of busy clinicians.

There was also agreement that the C-CES leader must strive to have and retain impartiality and an intellectual and emotional disengagement vis-a-vis the workplace where the C-CES is performed. This led the group to strongly opine that leaders ought not facilitate C-CES in clinical situations where they have also been clinically engaged. For the same reason, leaders should preferably not perform C-CES at their own workplace. Arguments included that coming to C-CES as a ‘stranger’ increases the likelihood of impartiality and not being burdened by pre-suppositions and makes it possible to ask “stupid questions” which may be beneficial to the C-CES process. The only negative aspect about coming from the outside, according to participants, was that this may mean some ethically salient aspects may be overlooked. However, it was pointed out that the risk of missing ethically salient aspects might be just as high when it comes to in-house C-CES, as one may become blind to familiar dilemmas. All in all, participants agreed that C-CES should preferably not be performed at the leader’s own workplace.

Regarding the C-CES leader’s role and approach, there was uniform agreement that the *facilitating* role, rather than adopting a leading or deliberating role, is most suited to C-CES work in the healthcare setting. Arguments included that the healthcare personnel should be facilitated to deliberate for themselves and come to their own conclusions in the case, and that this increases the chances for ethical learning and that what is decided during the C-CES is actually put into practice. Furthermore, that only healthcare personnel involved in patients’ care have the legal responsibility to make decisions with or on behalf of their patients. There was an animated discussion about whether the C-CES should be documented, in any form, in the patient’s medical file. Arguments against so doing included that knowing the C-CES would be documented might impact the discussion negatively, and that there was a risk that the documentation in the medical file might be erroneously interpreted as giving “the answer”. Arguments in favour of documentation included that it is of value to show that a C-CES has been conducted and to give guidance in pressing cases. Moderating comments included that documentation is only relevant in prospective cases (not retrospective), and that it is unclear by whom and what should be documented: that a C-CES has been performed, or relevant arguments brought forth, or a normative recommendation (or all of the above). On the topic of C-CES being seen as the final arbiter, participants uniformly agreed that the Swedish term “etisk rond” (English: “ethical round”) should be avoided and replaced by “etikrond” (English: “ethics round”) because although ethics is the *focus* in the C-CES, the C-CES format does not guarantee the ethicality of the whole process.

The normative discussion also identified several challenges in C-CES work. One related to the uptake of C-CES activities. It was mentioned that some workplaces work intensely with C-CES whereas others do not use C-CES at all, and that the variation does not mirror the level of ethical challenges at different workplaces. It was further mentioned that there is a lack of structures, at least in Sweden, for implementing and following up the recommendations generated during C-CES. The risk, then, is that the ethical potential of C-CES is underutilized. Also, the dominance of physicians in requesting C-CES was considered a problem, as the current power structures within healthcare might silence the voices of, for instance, assistant nurses.

## Discussion

In this paper, organizational and methodological aspects of C-CES interventions used in Swedish healthcare have been described and normatively discussed. Some results of this study are instantly recognisable from other descriptive studies. For instance many studies report the same time frames for C-CES (Magelssen et al., 2016; Molewijk et al., 2016), and similar recommendations to keep within strict time frames, to improve clinical uptake of C-CES (Bruun et al., 2019). Similarly, the eclectic skill mix of C-CES leaders match what is known from other countries (Schildmann et al., 2010).

As shown by the results there is great heterogeneity when it comes to the C-CES methods used in Sweden. This is in contrast with, e.g., Norway where there has been a national ambition to harmonize CES (Magelssen et al., 2016). The Norwegian model (The Six-Step Model) is used in Sweden also, but so is a Dutch model (the Dilemma method) as well as models developed in Sweden (the Actor model, the Karolinska model, and the "One to Five" method). As research has failed to establish the superiority of any particular C-CES method (Schildmann et al., 2019), the variation is not in itself alarming, and it may be more important that each CES organization and C-CES leader sticks to one or a limited number of methods to ensure consistent quality (Carrese et al., 2012). Considering the methodological variance, the *lack* of variation when it comes to the timeframes and goals of C-CES as well as the proper role of the C-CES leader becomes noteworthy. Indeed, our results indicate that the real-world difference between C-CES methods may be less important than perhaps intuited. The actual difference between the mentioned models as regards content and recommended steps in the process are minor.

The results suggest there may be some interesting “ought-is gaps” (Kuehlmeyer et al., 2024) in Swedish C-CES practice. First, the descriptive results show that many C-CES are performed by CES leaders in their own (clinical) working habitat, as has also been described in other countries (Magelssen et al., 2016). However, in the normative discussion there was agreement that this should be avoided, which is a line also taken in other normative papers on C-CES (Pfäfflin et al., 2009). Indeed, this study provides a new argument in this debate, in that the possibility to ask “stupid questions” may be an important upshot of coming to C-CES from the outside. Most C-CES leaders in the included organizations had a clinical background, which may make the challenges with being “too close” to the issues discussed during C-CES salient. At the same time, CES organizations rooted in a particular clinical setting may have loyalty and access issues which prevent them from performing C-CES activities outside their own clinical realm. This “ought-is gap” suggests it may be of value to develop models for exchange of CES services between clinical domains. Second, the level of ethical and methodological proficiency among Swedish C-CES leaders seems lower than recommended for instance in the US (Tarzian, 2013). In an era of “professionalization” and “standardization” of both clinical ethicists themselves and the clinical ethics consultations they perform (Mason, 2023; Spranzia, 2016), current Swedish practice seems somewhat conspicuous. At the same time, the jury is still out when it comes to the desired skill mix for C-CES leaders, and many texts advocate for non-theoretical competencies such as character traits, self-reflexivity and imagination in C-CES leaders (Friedrich, 2018; Slowther et al., 2004; Thornton, 2023), which this study did not assess.

The results indicate that C-CES are only rarely documented in Swedish patients’ medical records, and that Swedish C-CES leader are genuinely unsure of both the ethics and the practicalities of documentation. Whether and how to document C-CES has been highlighted as an important question (Yoon et al., 2020). Internationally there is a strong push for C-CES documentation in medical records (Eijkholt et al., 2022; Kaplan et al., 2022). There is no mention in Swedish jurisdiction on medical records when it comes to documentation of C-CES, nor are there any government guidelines on this topic. To the best of the authors’ knowledge, neither are any such guidelines immediately forthcoming. Hence, one implication of this study is that the Swedish CES community might take the initiative and investigate the question of documentation of C-CES.

It is also noteworthy that almost all respondents report using “exclusion criteria” to select cases deemed unsuitable for C-CES. However, some did not exclude themes and overarching questions and even ranked them as top three. It is disputable whether they really reported on C-CES, if it was not related to a specific case, or if “overarching questions” and “general themes” were dealt with through C-CES interventions in clinical cases. Furthermore, participants disagreed about the possible value of using hypothetical cases for C-CES, which most found unsuitable, whereas some found hypothetical cases relevant and sometimes rewarding. This mirrors the ongoing international debate about the optimal settings for C-CES (Jakobsen et al., 2024a). It could be argued that hypothetical cases work best when the aim is training or education, whereas the importance of using real cases grows the closer to clinical reality one comes. One reason is that specific and highly detailed facts of the case make a great difference when the aim is not only to identify important values but also to decide on actions/norms. Another reason is that healthcare personnel are probably more motivated to participate and also to follow up on the C-CES when it concerns a real, especially ongoing, patient case. Similarly to respondents in this study, previous C-CES literature also emphasises the importance of reserving the C-CES format for *ethical* challenges (Carrese et al., 2012; Førde & Pedersen, 2012; Molewijk et al., 2008; Tarzian, 2013). A previous Swedish study has argued that a pre-review of the case should be seen as an essential aspect of C-CES (Bartholdson et al., 2014). At the same time, many texts on C-CES emphasise that it may not always be obvious from beforehand whether the crux of the matter is indeed *ethical* or some other kind of challenge (Molewijk et al., 2011, 2016). It has also been questioned whether it is at all suitable to speak of any “crux of the matter” independent of the interpretation by, for instance, a C-CES leader (Agich, 2001). In light of this, it would be interesting to further investigate how “exclusion criteria” function in C-CES in Sweden and elsewhere.

The unfair distribution/uptake of C-CES in Swedish healthcare was normatively discussed and can be compared with the uptake in Norway where there are legal requirements for health trusts to have Clinical Ethics Committees (Magelssen et al., 2020). Furthermore, no respondent in this study reported that patients and/or families requested or attended C-CES. This is noteworthy as a previous Swedish study showed that healthcare personnel viewed patients’ and families’ perspectives as the second most important perspective in ethics discussions (Bartholdson et al., 2021). Patients’ and families’ participation is increasing in some other countries (Førde & Pedersen, 2011) and a research project, conducted in Nordic paediatric oncology, is exploring possibilities for patient and parent participation in C-CES, starting with the views of C-CES personnel (Billstein et al., 2025). The present study highlights the need to discuss the matter of patients’ possible representation in C-CES in the Swedish context.

### Strengths and Limitations

It must be pointed out that the descriptive part of this study had a small sample which could influence the representativeness to other CES organizations in Sweden and abroad. At the same time, the respondents together led or chaired a major part of the CES organizations in Sweden (for example all university hospitals in Sweden were included in one of the CES organizations), and their responses covered a large proportion of the C-CES provided in Sweden. The group of respondents also came from various professions, contexts, and geographical affiliations which likely further compensated for the sample size. Also, it could be considered a limitation that the key persons filled out the survey based on their own perceptions of the C-CES activities. Additionally, the researchers were familiar with the respondents and their C-CES, making the issue of bias a possible challenge.

Using a mixed-method design strengthens the study as it provides a more complete picture than a standalone quantitative or qualitative study. It also enhances the credibility of the results. Moreover, dividing the study into a descriptive and normative step made it possible to clarify and articulate standards that should guide further C-CES in Sweden and beyond.

### Suggestions for Further Studies

As suggested in the Discussion section, the question of “exclusion criteria” in C-CES merits further descriptive as well as normative inquiry. Some of the issues brought up by the normative discussion group also merit further considerations. The matter of follow-up after C-CES is a practical matter which can be studied empirically, either descriptively by looking at practices in other countries or by drawing up and implementing a programme for follow-up in Sweden. Furthermore, in a future study all the C-CES leaders (facilitators) in the country could be invited to respond to the survey.

As patients and families were not offered to request or participate in C-CES, unlike in other countries, there is a reason to discuss the Swedish C-CES culture in this aspect also. The issue of (un)equal access to C-CES by different professional groups could be studied using a descriptive survey, whereas the challenge of spreading C-CES beyond the workplaces which already have C-CES also involves tricky normative questions regarding “top-down” or “bottom-up” approaches to C-CES (Rasoal et al., 2017). Hence, although this study provides yet another puzzle piece on C-CES, the full puzzle is yet to be completed.

## Supplementary Information

Below is the link to the electronic supplementary material.

Supplementary Material 1

## Data Availability

Data will be made available upon reasonable request.
