# Supplementary material for: Case-Based Clinical Ethics Support – A Description and Normative Discussion of Methodological Issues from the Swedish Perspective
Source: HEC Forum. 2025 Oct 12;38(2):191–206. doi: 10.1007/s10730-025-09566-5 (PMC13194189; doi:10.1007/s10730-025-09566-5)
Supplement: Supplementary file 1 — Supplementary Material 1 [file 10730_2025_9566_MOESM1_ESM.docx]

Supplementary Information 1

**Descriptive Survey - Case-based Clinical Ethics Support (C-CES) in Sweden**

Please choose one C-CES activity (eg. Moral Case Deliberation (MCD), Ethics communication or Ethics round) that you/your team offer and fill in the information about that activity. If you offer several relevant C-CES activities, you will need to fill out a separate form for each type of activity. If you are several in this group of experts working together with the same method, you can fill this in together.

# Name of respondent(s):

# What do you call your C-CES activity?

For example, adapted Moral Case Deliberation (MCD)

# In what healthcare/care context do you perform this C-CES activity?

For example, paediatric oncology/hospital setting

# What would you say are the overall goals of the C-CES activity?

Highlight all alternatives that apply to your C-CES activity. If several alternatives apply, **please rank** them according to importance:

| Highlight all alternatives that apply  (Change the font colour to red): | Rank:  (Write number; most important =1) |
| --- | --- |
| - Enhance ethical competence/moral learning |  |
| - Decision making support |  |
| - Improving the quality of care |  |
| - Team building |  |
| - Promote ethical climate |  |
| - Providing protected space and time to voice ethical concerns |  |
| - Other, please specify­­­­­­­­­­­­­:­____________________ |  |

# What is the underlying theory of the C-CES activity?

For example, hermeneutics. Name the most important and provide a literature reference if available.

# What are the underlying normative assumptions?

These norms should apply to all participants in the C-CES activity (not just the ethicist/facilitator). Chose the two/three alternatives below most relevant to your C-CES

| Highlight two/three alternatives  (Change the font colour to red): | Rank:  (Write number; most important = 1) |
| --- | --- |
| - Listen, talk; try to understand; search ethics focus |  |
| - Clarify, ask questions; specify ethics focus |  |
| - Interpret, evaluate; change perspectives |  |
| - Analyse, argue, compare pros and cons |  |
| - Refer to, rely on values/norms |  |
| - Articulate problems (that are overlooked, neglected) or errors |  |
| - Apply, elaborate, conclude |  |
| - Suggest, recommend; respond to ethics focus |  |
| - Advocate, defend arguments, values or principles |  |
| - Insist on or resist against decisions or errors |  |
| - Other, please specify­­­­­­­­­: ____________________ |  |

# Organization

## 7a. Who can initiate the C-CES activity?

Highlight all alternatives below that apply to your C-CES activity. If several alternatives apply, **please rank** them according to who initiates your C-CES activity most often:

| Highlight all alternatives that apply  (Change the font colour to red): | Rank:  (Write number; most common =1) |
| --- | --- |
| - Reg. nurse |  |
| - Nursing assistant/Assistant nurse |  |
| - Physician |  |
| - Allied health personnel |  |
| - Psychologists/Psycho-social personnel |  |
| - Manager |  |
| - Patient |  |
| - Family member |  |
| - CES personnel (e.g. ethics committee member) |  |
| - Other, please specify­­­­­­­­­­­­­:­_______­­­______________ |  |

## 7b. What kind of ethical issues do you usually handle?

Highlight all alternatives below that apply to your C-CES activity. If several alternatives apply, **please rank** them according to frequency:

| Highlight all alternatives that apply  (Change the font colour to red): | Rank:  (Write number; most frequent =1) |
| --- | --- |
| - Prospective cases |  |
| - Retrospective cases |  |
| - General theme/topics (not related to a specific case, for example integrity) |  |
| - Overarching questions (at the management level, for example expensive drugs) |  |
| - Other, please specify­­­­­­­­­­­­­:­_____________________ |  |

## 7c. What, if any, exclusion criteria do you have for the ethical issues?

Highlight all alternatives below that apply to your C-CES activity (by changing font colour to red).

- - - Hypothetical case (participants do not have personal experience of the case)
    - Issues related to personnel (HR)
    - “Only” a problematic or distressful situation (not a moral question)
    - A theme (not a case)
    - Overarching questions (not a case)
    - Strong opinions rather than moral questions (not prepared to listen but rather using C-CES to convince others)
    - Other, please specify­­­­­­­­­­­­­

## 7d. Who usually participate in your C-CES activity?

Highlight all alternatives below that apply to your C-CES activity. If several alternatives apply, **please rank** them according to frequency:

| Highlight all alternatives that apply  (Change the font colour to red): | Rank:  (Write number; most frequent =1) |
| --- | --- |
| - - - Reg. nurse |  |
| - - - Nursing assistant/Assistant nurse |  |
| - - - Physician |  |
| - - - Allied health personnel |  |
| - - - Psychologists/Psycho-social personnel |  |
| - - - Manager |  |
| - - - Patient |  |
| - - - Family member |  |
| - - - CES personnel (e.g. ethics committee) |  |
| - - - Other, please specify­­­­­­­­­­­­­:­_____________________ |  |

## 7e. How is your C-CES activity scheduled?

Highlight all alternatives below that apply to your C-CES activity (by changing font colour to red):

- - - Acute
    - Regular
    - Other, please specify­­­­­­­­­­­­­

## 7f. How long time do you usually have for your C-CES activity?

Highlight the alternatives below that apply to your C-CES activity (by changing font colour to red):

- - - 30-60 minutes
    - 61-90 minutes
    - 91-120 minutes
    - Other, please specify­­­­­­­­­­­­­

# Structure the conversation in the C-CES activity?

## 8a. What is the status of the structure?

Highlight the alternatives below that apply to your C-CES activity (by changing font colour to red):

- - - Explicit structure
    - Implicit structure
    - Flexible structure, responsive to participants
    - Other, please specify­­­­­­­­­­­­­

## 8b. What method, if any, do you use to structure the conversation in the C-CES activity?

For example, the Dilemma method or the SME-method. Please also add a literature reference if available.

## 8c. If you have made any adaptations to the method, please describe:

# Characteristics of the people facilitating your C-CES activity

## 9a. What is the professional background of the facilitators?

For example, nursing

## 9b. What training do they have, if any?

Please describe, for example, facilitator training 3+2 days plus practice.

## 9c. What is the relationship of the facilitator with the participants in the C-CES activity?

Highlight all alternatives below that apply to your C-CES activity (by changing font colour to red):

- - - Inside (e.g. colleague at the same ward)
    - Outside (e.g. from another organization or ward)
    - Other:

# How do you document the performed C-CES activity?

Highlight all alternatives below that apply to your C-CES activity (by changing font colour to red):

- - - No documentation
    - Own documentation (available to CES personnel for example, the ethics committee)
    - Patient charts
    - Other:

**Thank you for completing this survey**!
